# Type I Interferon Response Is Delayed in Human Astrovirus Infections

**DOI:** 10.1371/journal.pone.0123087

**Published:** 2015-04-02

**Authors:** Susana Guix, Anna Pérez-Bosque, Lluïsa Miró, Miquel Moretó, Albert Bosch, Rosa M. Pintó

**Affiliations:** 1 Enteric Virus Group, Department of Microbiology, University of Barcelona, Barcelona, Spain; 2 Nutrition and Food Safety Research Institute (INSA-UB), University of Barcelona, Santa Coloma de Gramanet, Spain; 3 Digestive Physiology and Nutritional Adaptations Group, Department of Physiology, University of Barcelona, Barcelona, Spain; University of Tennessee Health Science Center, UNITED STATES

## Abstract

Type I interferon (IFN) activation and its subsequent effects are important in the response to viral infections. Here we show that human astroviruses (HAstVs), which are important agents of acute gastroenteritis in children, induce a mild and delayed IFN response upon infecting CaCo-2 cells. Although IFN-β mRNA is detected within infected cells and supernatant from infected cells show antiviral activity against the replication of other well-known IFN-sensitive viruses, these responses occur at late stages of infection once genome replication has taken place. On the other hand, HAstV replication can be partially reduced by the addition of exogenous IFN, and inhibition of IFN activation by BX795 enhances viral replication, indicating that HAstVs are IFN-sensitive viruses. Finally, different levels of IFN response were observed in cells infected with different HAstV mutants with changes in the hypervariable region of nsP1a/4, suggesting that nsP1a/4 genotype may potentially have clinical implications due to its correlation with the viral replication phenotype and the antiviral responses induced within infected cells.

## Introduction

Astroviruses were first identified by Appleton and Higgins in 1975, in association with an outbreak of infantile gastroenteritis in a maternity ward in England [1] and the *Astroviridae* family today contains viruses which infect more than 44 animal species [2]. Human astroviruses (HAstV) are recognized as common viral pathogens causing gastroenteritis in infants and young children, with very few reports of HAstV-mediated disease in normal healthy adults, and some reports of severe disease after dissemination to extra-intestinal tissues in immunocompromised patients [2,3]. Astroviruses are non-enveloped positive-strand RNA viruses containing a 6.8 kb polyadenylated genome linked to a VPg protein on the 5’end [3,4]. The genome contains three overlapping open reading frames (ORFs): ORF1a and ORF1b encode the viral protease and polymerase, respectively, and ORF2 encodes the capsid precursor [3]. The nonstructural proteins (nsPs) are translated from the genomic RNA as two large polyproteins, nsP1a and nsP1a/1b, through a translational frameshifting mechanism. Upon translation, nonstructural proteins participate in transcribing a full-length negative strand RNA, which serves as the template for the transcription of a new genomic and subgenomic RNAs. Subgenomic RNAs are then used to express capsid proteins. RNA transcription takes place in replication complexes assembled in close association with intracellular membranes which are thought to derive from the endoplasmic reticulum [5], and variability within the hypervariable region (HVR) contained in the C-terminal nsP1a protein (nsP1a/4) may affect the levels of genomic, subgenomic and antigenomic RNAs produced during infection, as well as the level of viral shedding in stools [6].

Despite the impact of astroviruses on human and animal health, very little is known about the mechanisms of pathogenesis [7,8], or the immune response to infection [8,9]. Both in humans and in some studied animals, astrovirus-infected intestines show relatively minor histological changes and inflammation, suggesting that major destruction of the intestinal epithelium and inflammatory responses do not play a role in AstV pathogenesis. Instead, it has been postulated that diarrhea may be caused by disruption of the absorptive/secretory function of the intestine and loss of intestinal epithelial barrier permeability [10,11,12]. In addition, the acute nature of the gastroenteritis, the short duration of symptoms, and the occurrence of most symptomatic infections in young individuals who may lack acquired immunity, strongly suggest that innate immune responses may play a key role in controlling virus replication and limiting disease in humans, especially in primary infections. Innate immune responses have been shown to contribute to the control of viral replication *in vivo* in infected turkeys and mice [13,14]. Interestingly, it has recently been demonstrated that complement factor C3 found on the surface of icosahedral viruses such as HAstVs can be internalized by the host cell and trigger cellular innate immune responses [15]. However, compared to other non-enveloped viruses, the level of complement-mediated innate immunity activation upon HAstV infection is substantially weaker, and this would be explained by the fact that the capsid protein of HAstV acts as a complement activation inhibitor [16,17,18].

The innate immune system forms the first line of defense against invading viruses, limiting initial virus replication and ensuring survival of the host until a full, specialized adaptive response is developed. Type I interferons (IFNs) are secreted key cytokines that protect uninfected cells and stimulate leukocytes acting at the interface of innate and adaptive immunity [19]. Tissue cells recognize invading viruses mainly by intracellular pathways [20]. In the cytoplasm, some viral molecules such as double-stranded RNA (dsRNA) or RNAs bearing a 5’-triphosphate group are detected by cellular pathogen recognition receptors (PRRs) such as RIG-I (retinoic acid-inducible gene 1) or MDA5 (melanoma differentiation-associated protein 5). These activated receptors trigger a signaling cascade that activates MAVS (mitochondrial antiviral-signaling protein) and results in the phosphorylation of the transcription factor IRF-3, a member of the IFN regulatory factor (IRF) family. Phosphorylated IRF-3 moves into the nucleus where promotes transcription of the IFN-β promoter. Secreted IFN-β binds to the type I IFN receptor which is present on virtually any host cell and activates IFN signaling via the JAK/STAT (Janus kinase/Signal transducer and activator of transcription) pathway, resulting in the activation of more than 300 IFN-stimulated genes (ISGs) which inhibit virus multiplication at the level of transcription, translation, genome replication, assembly, and exit, and stimulate the subsequent adaptive immune responses. Virtually all viruses studied to date have developed means to counteract or minimize the induction, signaling, or antiviral actions of the IFN responses [19,21].

In this study, we have examined the type I interferon response and status of IRF-3 following infection of the intestinal carcinoma cell line CaCo-2 with HAstV. Our results indicate that infection induces an attenuated type I IFN response during the late steps of viral replication. Knowledge of the host response to HAstV may provide keys for prevention and treatment of the human disease.

## Materials and Methods

### Cells and viruses

CaCo-2 (ECACC 86010202), HeLa (ATCC CCL2), MA-104 (ATCC CRL-2378.1), and Vero (ATCC CCL81) cells were grown in Eagle’s minimum essential medium (MEM) supplemented with 10% fetal bovine serum (FBS). A cell culture-adapted strain of serotype 4 HAstV (kindly provided by WD Cubitt from the Great Ormond Street Hospital, London), as well as three serotype 1 HAstV mutants differing in their nsP1a/4 gene (HAstV-IV, HAstV-VI and HAstV-XII) [6] were used in this study. Propagation and preparation of HAstV stocks was performed as previously described [4]. Briefly, viruses were pretreated with 10 μg of trypsin (GIX Sigma) per ml for 30 min at 37°C and were diluted in MEM 0% FBS in order to obtain the multiplicity of infection (MOI) required for each experiment. CaCo-2 cell monolayers were washed twice with MEM 0% FBS and inoculated with the virus. After a 1-h adsorption at 37°C, cells were washed once and MEM 10% FBS was added in order to ensure a single cycle of viral replication. Infectious titers of stocks were determined by a cell-culture RT-PCR method previously described [22], and by immunofluorescence analysis of infected monolayers. Encephalomyocarditis virus (EMCV) and rotavirus (RV) SA11 (ATCC VR-899) were grown on HeLa and MA-104 cells, respectively, and infectious viral titers were measured by TCID50 assays on Vero cells and MA-104 cells, respectively.

### Virus inactivation

HAstV stocks were inactivated by incubation for 1 h at room temperature in a class II biological safety cabinet under the UV light. Complete viral inactivation was confirmed by lack of capsid protein expression by immunofluorescence after infecting CaCo-2 cells.

### Reagents and antibodies

The synthetic analog of dsRNA polyinosine-polycytidylic acid (polyI:C) and the BX795 inhibitor were purchased from InvivoGen. BX795 was dissolved in DMSO and stored as a 10 mM solution at -20°C. Recombinant human IFN type I (human interferon-αA/D) was purchased from Sigma Aldrich. MAb 8E7 specific against the capsid protein of HAstV was kindly provided by R-Biopharm AG. Polyclonal antibody against HAstV (Rab3) was kindly provided by Dr DM Bass from the Department of Pediatrics, Stanford University, USA. Rabbit polyclonal IRF-3 was purchased from Santa Cruz Biotechnologies. Cy3 labeled goat anti-mouse IgGs (Amersham GE Healthcare) and Alexa 488 labeled anti-rabbit IgGs (Sigma) were used as secondary antibodies for immunofluorescence analysis.

### RNA isolation, RT-PCR and quantitative real-time RT-PCR (qRT-PCR)

Total RNA from cells was prepared using TRIzol LS reagent (Invitrogen) following the manufacturer’s instructions. To remove any traces of contaminating DNA, extracted RNA was treated with 1 U of RQ1 RNase-free DNase (Promega) for 1 h at 37°C and DNase was heat inactivated 10 min at 65°C. Conventional RT-PCR reactions to detect HAstV RNA, IFN-β, GAPDH (glyceraldehyde-3-phosphate dehydrogenase), and ISG56 mRNAs were performed using Expand Reverse Transcriptase and Expand High Fidelity PCR System (Roche). HAstV genome amplification was performed using primers A1 and A2 as described previously [23,24]. IFN-β, GAPDH, and ISG56 mRNA amplification was performed using primers described elsewhere [25,26].

Quantitative real-time RT-PCR assays were carried out using a Mx3000P instrument (Stratagene). Primers and probes for HAstV genome quantification target ORF1b region and have been previously described [27]. Standard curves for HAstV RNA quantification were included in every assay and were generated by using pAVI6 plasmid, which contains the complete genome of HAstV-1 (Accession number L23513, kindly provided by Dr. SM Matsui, VA Palo Alto Health Care System, Palo Alto, USA) [28]. For quantification of IFN-β and GAPDH mRNAs, primers and probes were designed using the Real-Time PCR Assay Design Tool from Integrated DNA Technologies (IDT) website. IFN-β specific primers and probe were 5’ AAG GCC AAG GAG TAC AGT-3’, 5’- CAC AGG CTA GGA GAT CTT CA-3’, and 56-FAM/TAG GAT TTC CAC TCT GAC TAT GGT CCA GG/3IABlk_FQ. GAPDH specific primers and probe were 5’- ACA TCG CTC AGA CAC CAT-3’, 5’- GGG TCA TTG ATG GCA ACA-3’, and 56-FAM/ACC AAA TCC GTT GAC TCC GAC CTT /3IABlk_FQ. For IFN-β and GAPDH quantification, a standard curve was constructed in every assay using 5 10-fold serial dilutions of the RNA sample corresponding to the positive and negative controls of the experiment, respectively. GAPDH mRNA titers were used as an endogenous control to normalize all samples versus the number of cells. All samples were quantified at least in duplicate. Reactions were performed using the One step RT qPCR MasterMix Plus kit (Eurogentec). The thermal protocol consisted of 30 min at 48°C, followed by 10 min at 95°C and 40 cycles of 15 s at 95°C and 1 min at 60°C.

### Immunofluorescence (IF) assays

Virus-infected CaCo-2 cells grown on glass coverslips in 24-well plates were rinsed twice with phosphate buffered saline (PBS) and stained by immunofluorescence as previously described [4]. Samples were analyzed under a fluorescence microscope (Leica DMRB FLUO). Mean percentage of infected cells was calculated from 5 fields of each coverslip, after analyzing images using ImageJ software [29].

### Measurement of antiviral activity by a virus infectivity reduction bioassay

The amount of antiviral activity secreted by cells was estimated by using a bioassay in HeLa cells. Before performing the assay, supernatants of HAstV-infected or transfected cells were UV-inactivated. HeLa cells grown on 96-well plates were pretreated with 2-fold dilutions of inactivated supernatant samples for 24 h at 37°C, before infection with EMCV at a MOI of 1 TCID50/cell. The development of EMCV-induced cytopathic effect (CPE) was monitored at 48 h post-infection (hpi). Cells pretreated with 2-fold dilutions of culture media containing 1,000 U/ml of recombinant type I IFN were used as a reference control, and levels of antiviral activity were expressed relative to it.

### ELISA assay for quantification of HAstV capsid protein

An indirect enzyme-linked immunoassay was performed on serial 4-fold dilutions of samples. Astrovirus antigens in cell lysates were captured with mAb 8E7 and detected by using a rabbit polyclonal anti-HAstV antibody (Rab-3). Specifically bound antibodies were revealed by a peroxidase-conjugated anti-rabbit IgG antibody (Sigma Aldrich). Undiluted cell lysates from mock-infected cultures were used as negative controls to calculate a positivity cutoff value (mean + 3 standard deviations). Sample dilutions which had an OD value higher than the cutoff value were considered positive.

### Measurement of transepithelial resistance of CaCo-2 cell monolayers

CaCo-2 cells were differentiated for 21 days on 1.12-cm^2^ semipermeable tissue culture inserts (0.4 μm pore size, Corning Incorporated), with cell culture medium renewal on alternate days. Transepithelial resistance (TER) was confirmed to be at least 1,000 Ωxcm^2^ using an EVOM voltometer (World Precision Instruments, Sarasota, FL) before infection, and was monitored every 4–12 hours during the course of infection. Results were presented as percentages of the insert’s TER reading versus 0 hpi.

### Statistical analysis

Comparisons between means were performed using the student t-test (unpaired) using the IBM SPSS Statistics version 20 software (SPSS Inc., Chicago, IL, USA). P values < 0.05 were considered statistically significant.

## Results

### HAstV infection induces a weak type I IFN response

**Fig 1:**
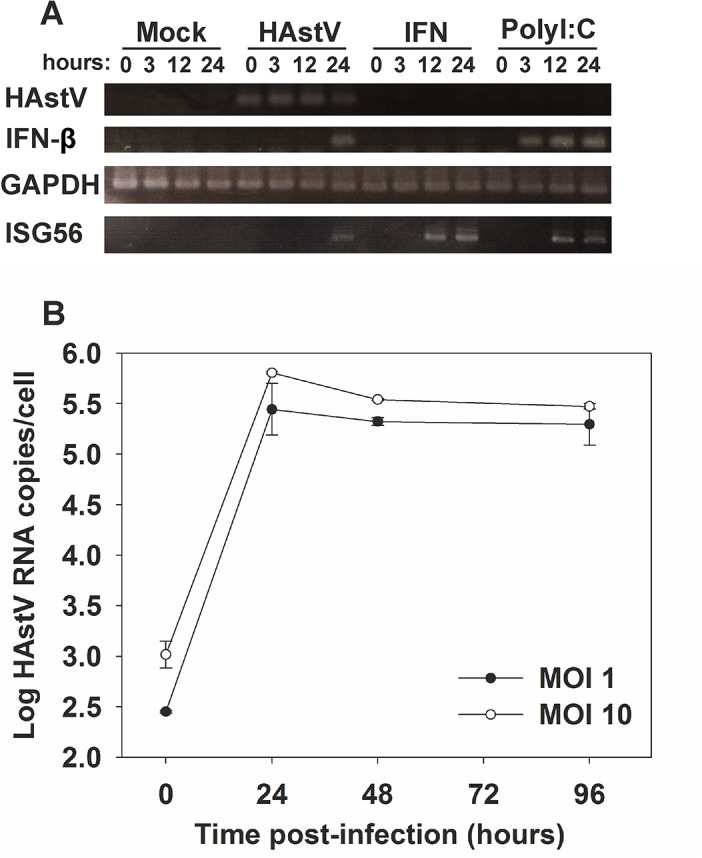
Induction of an IFN response is delayed during HAstV infection. (A) Temporal analysis of induction of IFN-β and ISG56 mRNA expression by in CaCo-2 cells infected with HAstV at a MOI of 1. Mock-infected cells, cells treated for 24 h with exogenous IFN at 1,000 U/ml, and polyI:C-transfected cells were used as controls. (B) HAstV growth curve on CaCo-2 cells at 2 different MOIs. Total HAstV RNA was measured by qRT-PCR at the indicated times post-infection. Data represent mean values of duplicate wells and error bars represent the standard error of the mean (SEM).

To examine whether HAstV infection induces an IFN response, CaCo-2 cells were infected at a MOI of 1 and RNA was extracted at 0, 3, 12 and 24 hpi (Fig 1A). Mock-infected cells, cells transfected with polyI:C and cells treated with 1,000 U/ml of recombinant type I IFN were used as controls. Conventional RT-PCR was performed to detect viral RNA, IFN-β mRNA, and ISG56 mRNA. GAPDH mRNA was amplified as a quality control for RNA. Results show that polyI:C transfection induced IFN-β gene transcription as early as 3 hours post-transfection (hpt), while IFN-β mRNA could not be detected in HAstV-infected cells before 24 hpi. A time course analysis of HAstV RNA synthesis was performed by qRT-PCR after infecting cells at 2 different MOIs. As expected, a 3-log increase in viral RNA titers was observed during the first 24 hours for both MOIs (Fig 1B). Together, these results suggest that HAstV delays the onset of IFN induction sufficiently until a large amount of progeny particles are produced.

Expression of ISG56 mRNA accumulated in cells treated with exogenous IFN and polyI:C-transfected cells starting at 12 h (Fig 1A). Detection of ISG56 mRNA in HAstV-infected cells at 24 hpi could indicate that IFN-β had already been released to the extracellular media, although it has also been described that some viruses from diverse families can induce synthesis of ISG56 mRNA in the absence of both IFN production and/or virus replication [26,30].

**Fig 2:**
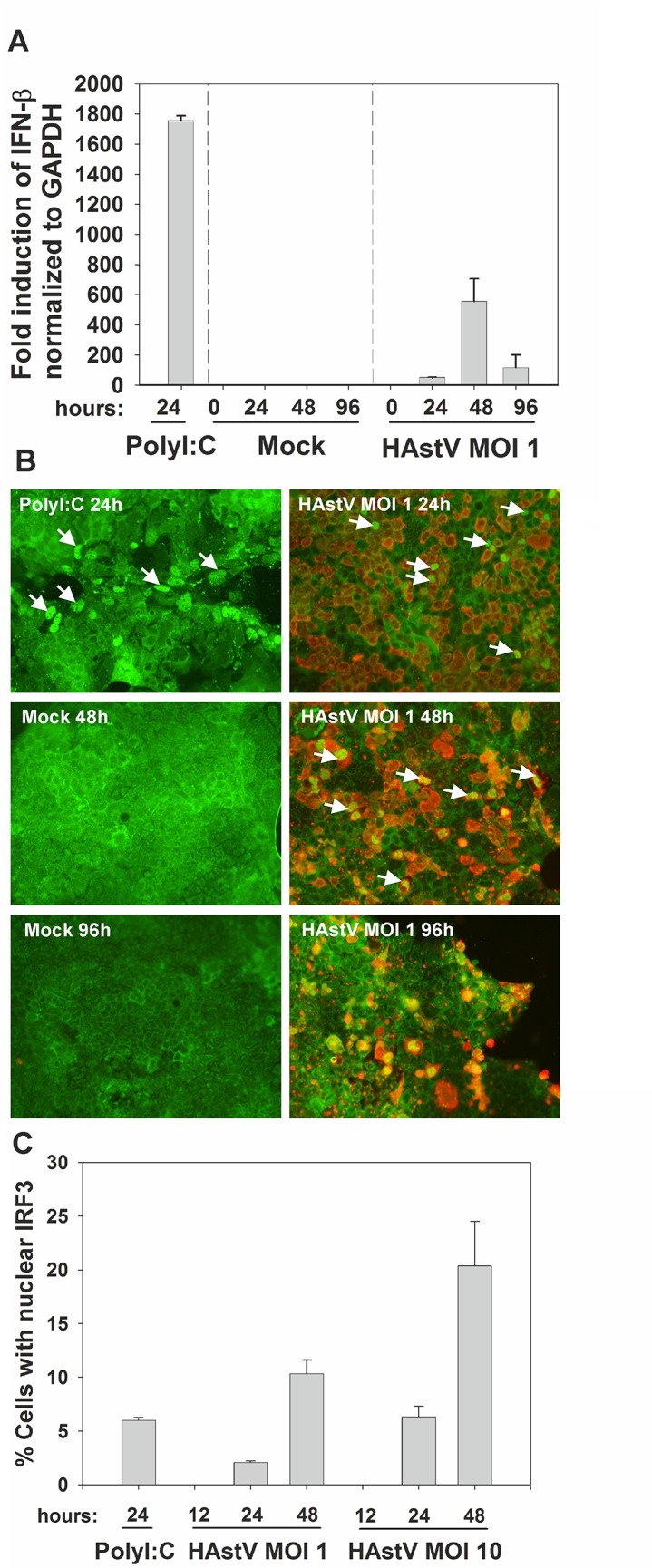
Analysis of the level of IFN response in HAstV-infected cells. (A) Quantification of IFN-β mRNA levels by qRT-PCR during infection of CaCo-2 cells at a MOI of 1. qRT-PCR values with primers specific for human IFN-β mRNA were normalized to endogenous GAPDH mRNA levels at each time point, and results were expressed as fold induction of IFN-β expression versus 0 hpi. PolyI:C-transfected cells at 24 hpt were used as a positive control. Results shown are the mean values of 2 independent experiments and error bars represent the SEM. (B) Kinetic analysis of IRF3 subcellular localization during HAstV infection at a MOI of 1. PolyI:C transfected cells fixed at 24 h post-transfection and mock-infected cells fixed at 48 and 96 hpi were used as positive and negative controls, respectively. Cells were labeled for HAstV capsid protein (red) and IRF3 (green). White arrows indicate cells with nuclear translocation of IRF3. (C) Percentage of cells with translocation of IRF3 into the nucleus. Data (mean values ± SEM) were calculated after counting the number of cells with nuclear IRF3 from 5 fields from coverslips from 2–3 independent experiments using the Image J software.

In order to analyze the magnitude of the IFN response, levels of IFN-β mRNA were quantified by qRT-PCR, and subcellular localization of IRF3 was examined by immunofluorescence during the course of infection at a MOI of 1, which results in more than 90% of infected cells as measured by IF. IFN-β mRNA levels induced by HAstV peaked at 48 hpi but were 3.2-fold lower than those induced by polyI:C transfection (Fig 2A). Since transfection efficiency of CaCo-2 cells is low and polyI:C transfection only resulted in 6 ± 0.7% of cells with nuclear IRF3 producing IFN-β mRNA (Fig 2C), when comparing the total levels of IFN-β mRNA produced by polyI:C-transfected cultures to the levels produced by infected cultures, it would seem that in these latter cultures, either each infected cell produced very low levels of IFN-β mRNA or that IFN-β mRNA would only be produced by a few number of infected cells. Co-staining of viral structural proteins and IRF3 showed that only 2 ± 0.2% of HAstV-infected cells had nuclear IRF3 at 24 hpi (Fig 2B and 2C). At 48 hpi, the percentage of cells with nuclear IRF3 increased up to 10.34 ± 1.8 0%, but in any case was not close to 100%. Percentages were higher when infecting cells with a MOI of 10, with a maximum of 20.4 ± 5.8% at 48 hpi (Fig 2C). At 96 hpi, cell damage due to infection was too strong to allow proper interpretation of IF data (Fig 2B). At 12 hpi, IRF3 was localized to the cytoplasm of all HAstV-infected cells (data not shown). Together these results indicate that, despite the high number of HAstV-infected cells, the IFN response in infected cultures was attenuated.

**Fig 3:**
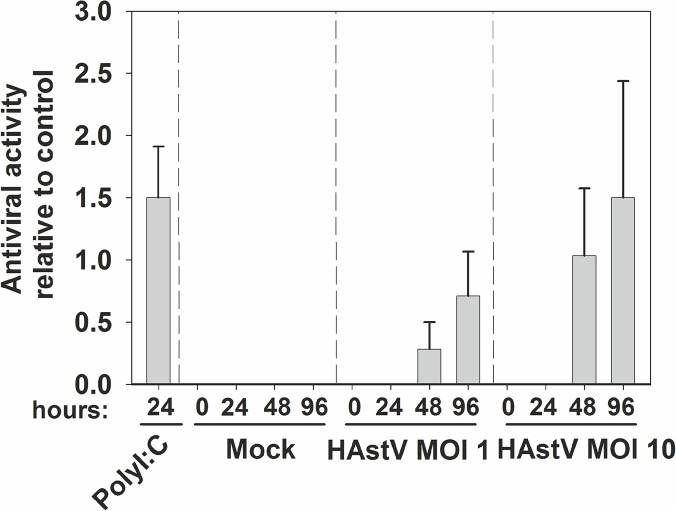
Level of antiviral activity released into the supernatant during HAstV infection. Antiviral activity in the supernatant of poly:IC-transfected cells, mock-infected cells and HAstV-infected cells at a MOI of 1 and 10 was measured by a virus infectivity reduction bioassay using EMCV, which is sensitive to IFN. HeLa cells were treated for 24 hours with serial 2-fold dilutions of the indicated supernatant, and were infected with EMCV. Results expressed as level of antiviral activity relative values to the reference control (cells treated with 1,000U/ml of recombinant type I IFN) are shown. Data are pooled from three independent experiments and error bars represent the SEM.

To determine if transcription of IFN-β induced by HAstV could result in production and cell release of type I IFN, we measured the presence of antiviral activity in supernatants from HAstV-infected cells at two different MOIs, using a virus infectivity reduction bioassay, using treatment with 1,000U of type I IFN as a reference control (Fig 3). In order to make sure that no residual HAstVs would remain in the supernatants, samples were inactivated by a 1-h UV incubation prior to the assay. Total viral inactivation was confirmed by lack of positive cells by IF analysis. Antiviral activity against EMCV could be detected in supernatants of HAstV-infected cells starting at 48 hpi, suggesting that activation of IFN-β gene results in protein production and secretion to the extracellular environment.

### IFN response to HAstV is dependent on virus replication

**Fig 4:**
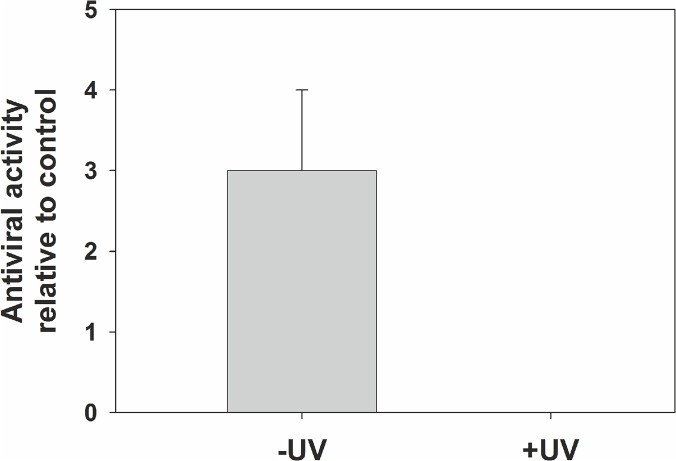
HAstV replication is essential for induction of a cellular antiviral response. Antiviral activity in the supernatant of cultures infected with HAstV at a MOI of 10 (or an equivalent amount of UV-inactivated virus), and harvested at 48 hpi, was measured using a virus infectivity reduction bioassay. Results are expressed as level of antiviral activity relative values to the reference control (cells treated with 1,000U/ml of recombinant type I IFN). Data represent mean values of 2 independent experiments and error bars represent the SEM.—UV: Non-inactivated virus; +UV: Inactivated virus.

To determine if productive viral replication was required for induction of an innate response, CaCo-2 cells were inoculated with infectious HAstV or an equivalent amount of UV-inactivated virus (Fig 4). No differences were observed in the viral dose as confirmed by RT-qPCR. Antiviral activity in the supernatant of cultures was never observed in cells infected with UV-inactivated viruses, even at higher MOIs (data not shown), indicating that viral replication is essential for cells to activate an antiviral response.

### Inhibition of cellular IFN response enhances HAstV replication

In order to determine whether blocking of intracellular IFN defense mechanism benefits HAstV replication, the effect of treatment of CaCo-2 cells with BX795 inhibitor was examined. BX795 is an aminopyrimidine compound which inhibits the catalytic activity of TBK1/IKKε (TANK-binding kinase 1/IkappaB kinase epsilon) by blocking its phosphorylation [31]. CaCo-2 wells were pre-treated with 10 μM BX795 (or DMSO for untreated controls) for 3 hours at 37°C before infecting them with a MOI of 10. After infection, DMSO (untreated), 1μM or 5 μM of BX795 was added in the culture media, and HAstV production was measured by quantification of viral RNA by qRT-PCR (after normalization versus GAPDH mRNA levels), assessment of infectious viruses released in the supernatant, and measurement of HAstV capsid protein in total cell lysates by ELISA. Lack of cellular toxicity was estimated by observation of cells under the microscope and by detection of unchanged levels of GAPDH mRNA by qRT-PCR in all BX795-treated cells, and absence of cellular IFN responses were confirmed by the lack of expression of IFN-β mRNA by qRT-PCR (data not shown).

Results show that treatment of cells with 5 μM of BX795 resulted in a significant 2-fold increase in total HAstV RNA produced as well as in the amount of infectious progeny released in the supernatant compared to untreated cells (Table 1). However, quantification of total capsid protein by ELISA indicated a 4-fold increase compared to untreated cells, suggesting that although a higher amount of viral capsid proteins were produced, only half of these proteins were able to be fully converted into infectious virions.

**Table 1:** Fold change in HAstV progeny on CaCo-2 cells treated with BX795 versus untreated cells (results are representative of 2 independent experiments, showing mean values ± standard error of the mean).

	Untreated	1 μM BX795	5 μM BX795
Total HAstV RNA (qRT-PCR)	1 ± 0.8 *	1.60 ± 0.03	2.03 ± 0.25
Released infectious viruses (infectivity)	1 ± 0.10	1.28 ± 0.06	1.93 ± 0.15 *
Total HAstV capsid protein (ELISA)	1 ± 0.0	1 ± 0.0	4 ± 0.0 *

Asterisks indicate statistically significant differences (p<0.05).

### Exogenous IFN inhibits viral replication

**Fig 5:**
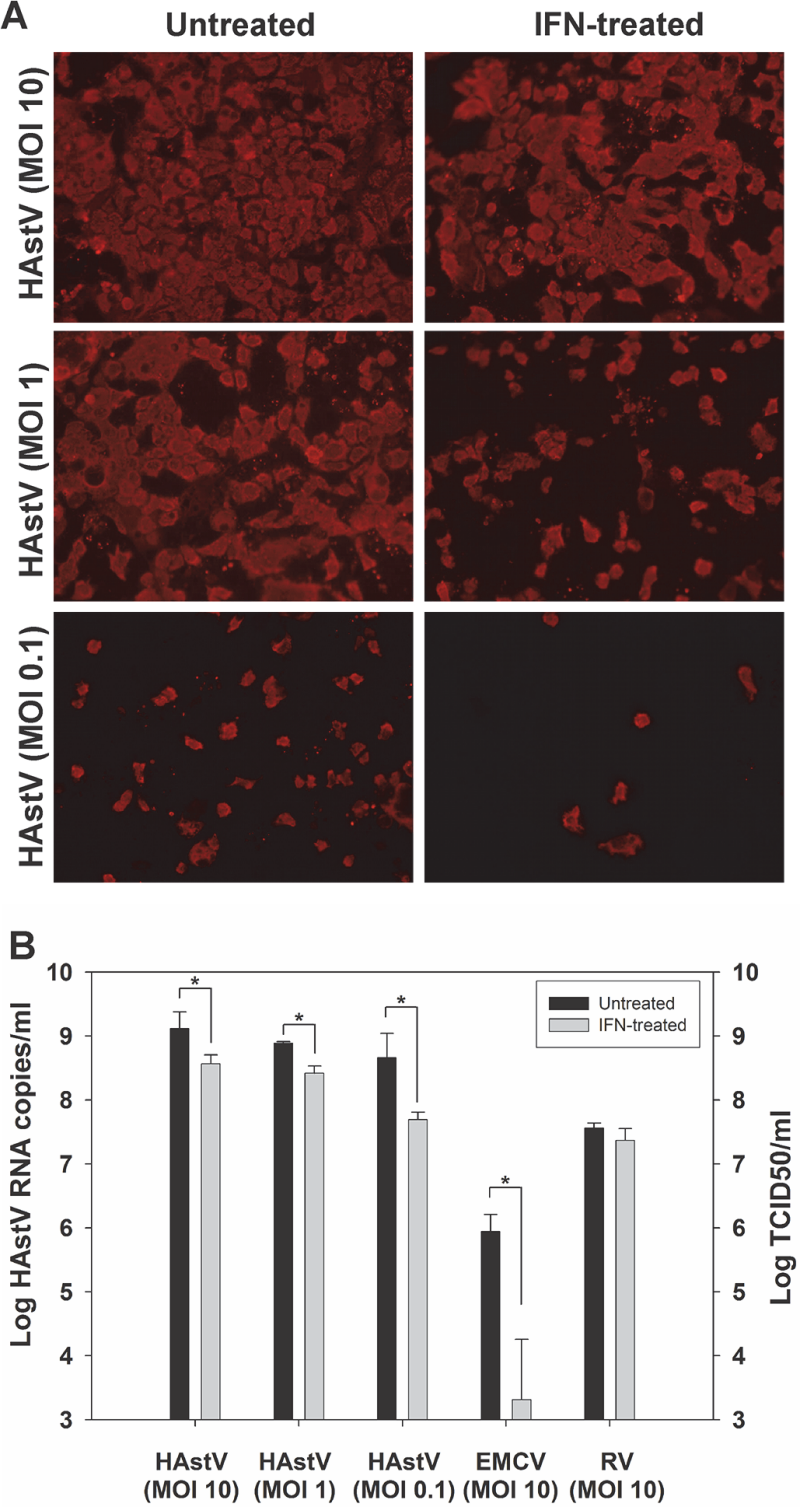
Effect of exogenous IFN on HAstV replication. (A) IF analysis of CaCo-2 cells after a 24 h pre-treatment with 1,000 U IFN/ml and infection at different MOIs. (B) Quantitative measurement of HAstV, EMCV, and RV progeny release in the supernatant of untreated cells and IFN-treated cells. Data represent mean values of 2–3 independent experiments and error bars represent the SEM. Asterisk indicates a statistically significant difference between mean titers from untreated and IFN-treated samples (t-test) (p<0.001).

In order to understand whether HAstV replication is sensitive to IFN, CaCo-2 cells were pre-treated with 1,000 U/ml of type I IFN for 24 hours before infection at three different MOIs. EMCV and and RV SA11 were used as controls for an IFN-sensitive virus and an IFN-resistant virus, respectively. The number of HAstV-infected cells observed by IF at 24 hpi was slightly reduced after IFN treatment (Fig 5A). Virus progeny released into the supernatant was measured for HAstV by qRT-PCR. EMCV and RV yields were measured by TCID50 titration in Vero and MA-104 cells, respectively. Reduction of HAstV replication after IFN pre-treatment was significant in all cases (p<0.001), but on average, there was a reduction of 0.8 ± 0.2 log, which was less than what was observed with EMCV (Fig 5B). As expected, RV was not affected by IFN-treatment.

### HAstV infection is not able to block IFN response induced by dsRNA

In order to examine whether infection with HAstV inhibits the ability of cells to produce IFN-β in response to treatment with polyI:C, CaCo-2 cells were mock-infected or infected with HAstV at a MOI of 1, and transfected with polyI:C at 8 hpi. Twenty-four hours later, total RNA was analyzed by qRT-PCR to determine the level of IFN-β mRNA induction, and supernatant of cells was collected to measure antiviral activity using the virus infectivity reduction bioassay. CaCo-2 cells infected with rotavirus at a MOI of 5, which is known to block the IFN response [32,33], were used as a positive control.

**Fig 6:**
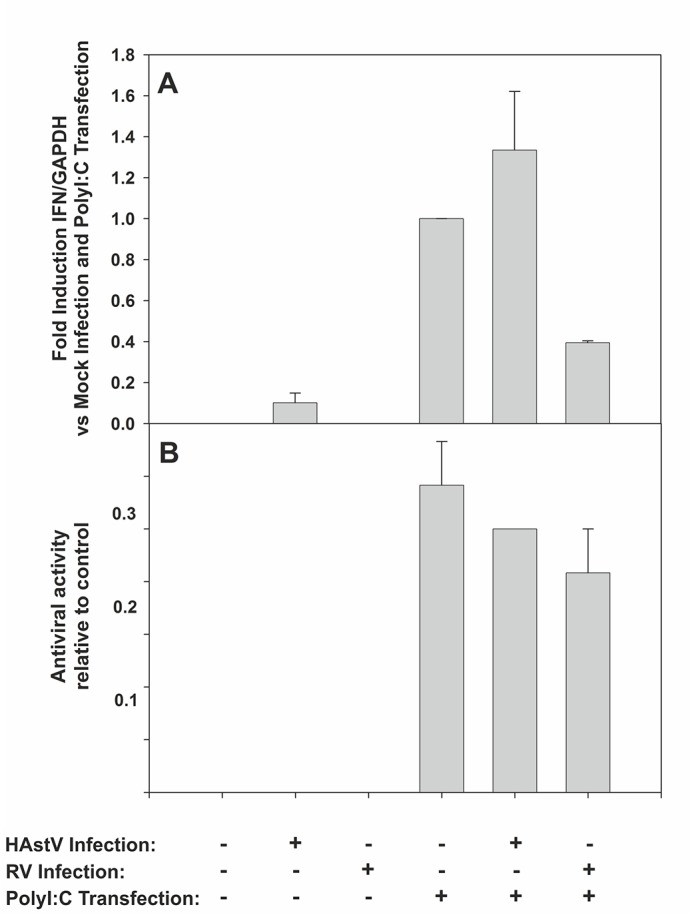
HAstV is not able to inhibit the IFN response induced by polyI:C transfection. CaCo-2 cells were either mock-infected, infected with HAstV at a MOI of 1 or RV at a MOI of 5, and 8 hours later, they were mock-transfected or transfected with polyI:C. (A) Level of IFN-β mRNA normalized versus GAPDH produced within each experimental condition at 32 hpi. (B) Level of antiviral activity in the supernatant of cultures at 32 hpi. Results are expressed as relative values to the reference control (cells treated with 1,000U/ml of recombinant type I IFN). Data represent mean values of 2 independent experiments and error bars represent the SEM.

Results suggest that HAstV infection is not able to disrupt the innate immune sensing pathway induced by polyI:C (Fig 6). Only a previous infection with RV was able to reduce by 60% the IFN-β mRNA levels produced after polyI:C transfection, although differences were not statistically significant (Fig 6A). HAstV and RV yields were similar between mock-transfected wells and wells transfected with polyI:C (data not shown). As expected, antiviral activity in the supernatant of cultures at 32 hpi could only be detected in cells transfected with polyI:C, and the response could only be reduced by the presence of rotavirus infection (Fig 6B).

### Higher percentage of infected cells correlates with higher levels of transepithelial resistance (TER) disruption and higher levels of IFN-β response, but type I IFN does not cause a change in the TER

**Fig 7:**
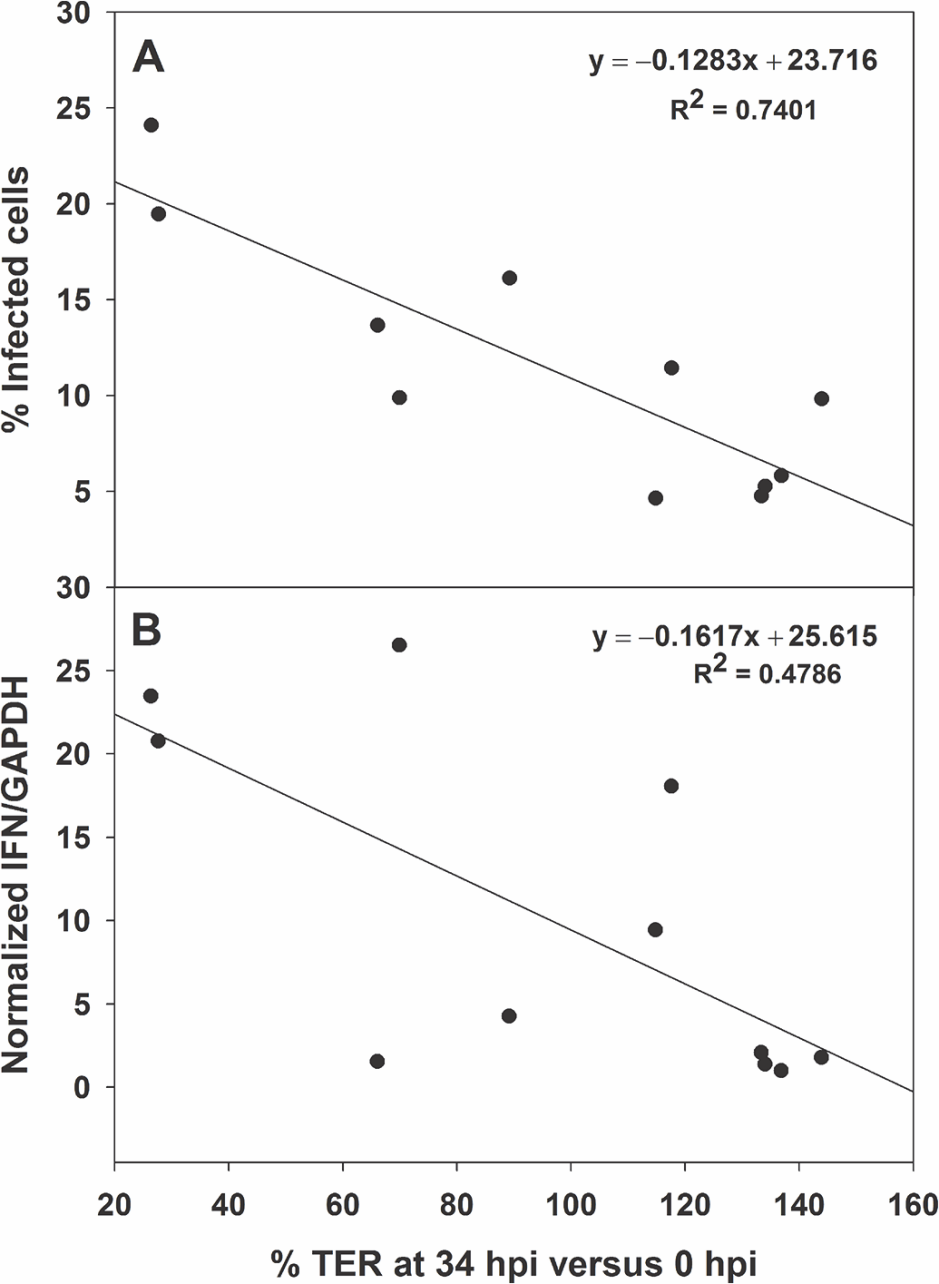
Correlation analysis between the reduction in transepithelial resistance (TER) and the percentage of infected cells (A), and the level of IFN-β response (B). TER levels are expressed as the percentage of the measurement at 34 hpi versus the value at 0 hpi. Percentages of infected cells were expressed as the average percentage of IF-positive cells after analyzing 5 fields of each coverslip. Level of IFN-β response was measured by qRT-PCR from total cellular RNA and normalized for GAPDH levels. Results are expressed relative to the lowest IFN-β response level, which was considered 1. A total of 11 pairs of samples were collected from 3 independent experiments.

Since it has been described that HAstV infection increases barrier permeability on differentiated CaCo-2 cells [10], we examined whether there was a correlation between disruption of intestinal barrier, the number of infected cells and the IFN-β response. Cells were differentiated and polarized on semipermeable inserts and infected apically at a MOI of 2 or 10. In these experiments, virus inoculum was not activated with trypsin in order to preserve cell monolayer. Activation of IFN-β response was measured by qRT-PCR and normalized versus GAPDH levels at 24 hpi on total cellular RNA as above described, and TER levels were measured every 4–12 h through 34 hpi. The percentage of infected cells was calculated in each well by an immunofluorescence assay and image analysis of 5 fields. It did not reach 100% in any case, even when using a MOI of 10, probably due to the fact that viruses were not pre-activated with trypsin before infection. As shown in Fig 7A, significant reductions in TER levels were only achieved in wells where more than 10% of cells in the monolayer were infected, and a positive correlation was also observed between percentage of TER reduction and level of IFN-β response (Fig 7B). However, incubation of cells with 1,000 U/ml of type I IFN did not affect TER, suggesting that increase in cellular permeability would be caused by a viral component or by a cellular factor different from type I IFN.

### Different viral strains induce different levels of IFN-β response

**Fig 8:**
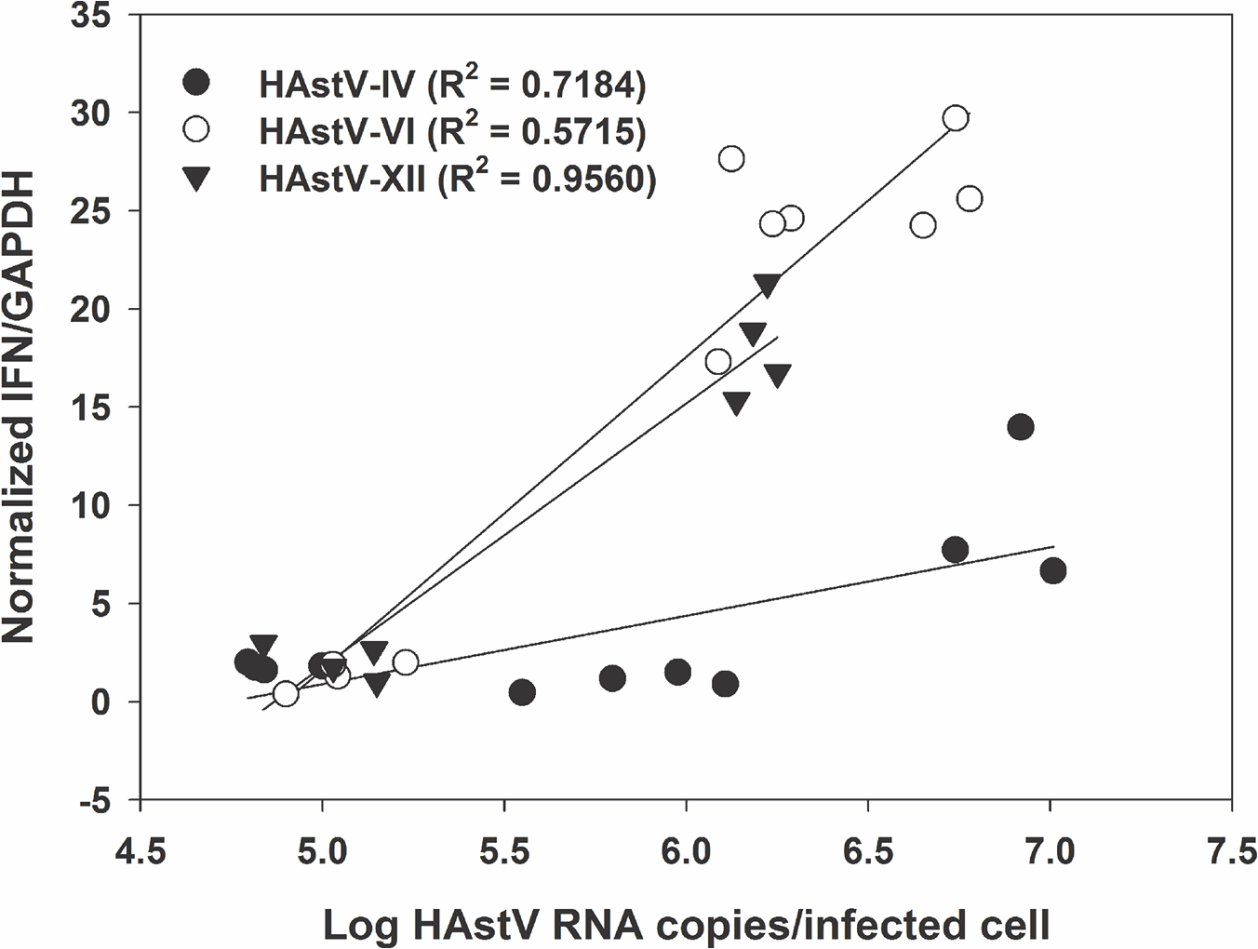
Effect of nsP1a/4 genotype on the level of IFN-β response. Normalized levels of IFN-β/GAPDH response at 24 hpi were plotted against the average number of HAstV genome copies per infected cell after infecting differentiated CaCo-2 cells with different nsP1a/4 mutants at the same MOI. Data summarize results from three independent experiments.

Since viruses containing different nsP1a/4 variants have been associated with higher replication phenotypes and higher levels of virus shedding in stools, we evaluated whether nsP1a/4 variability influences the magnitude of the IFN-β response. CaCo-2 cells were infected at a MOI of 2 or 10 with different HAstV mutants differing in their nsP1a/4 HVR. IFN-β activation was measured by qRT-PCR and normalized versus GAPDH, and the average number of viral genome copies per infected cell was estimated after quantifying total HAstV RNA by qRT-PCR and normalizing it by the number of infected cells within each well. The average number of log HAstV genome copies per infected cell was, 5.8±0.9, 5.9±0.7, and 5.6 ± 0.6 for HAstV-IV, HAstV-VI and HAstV-XII, respectively. Results shown in Fig 8 indicate differences between HAstV mutants in terms of IFN-β activation. The level of IFN-β response in cells infected with HAstV containing nsP1a/4 genotype IV was lower than the IFN response induced by mutants HAstV-VI and XII, especially when the number of HAstV genomes per infected cell was high.

## Discussion

In this work, we have examined the type I IFN response and the status of IRF3 in HAstV-infected CaCo-2 cells. Our findings demonstrate that HAstV infection does not induce a strong type I IFN response, as evidenced by the fact that IFN-β mRNA is only detected once viral replication has taken place and at low levels. When comparing the levels of IFN-β mRNA produced in infected cultures with a high proportion of infected cells, with those produced in polyI:C-transfected wells, it seems that either each infected cell produces very low levels of IFN-β mRNA or that only a low proportion of infected cells produce a higher amount of IFN-β mRNA. Consistent with this latter idea, only a low percentage of infected cells show translocation of IRF3 to the nucleus, suggesting that IFN induction does not take place in each infected cell. Although it cannot be completely ruled out that IFN-β synthesis may occur independent of IRF3 activation, our working hypothesis is that IFN-b mRNA would not be produced within every infected cell. In addition, nuclear translocation of IRF3 occurs at late times post-infection, and antiviral activity in the supernatant of infected cells is not detected until 48 h after infection, confirming that in any case, IFN production takes place at the late stages of the viral replication cycle and requires infectious viruses. Finally, when we examined the ability of HAstV to inhibit the IFN response induced by polyI:C transfection, we observed that HAstV infection is not able to block the ability of the cell to respond to dsRNA as it has been observed for other well-studied ssRNA viruses [21]. HAstV behavior would be more similar to what has been described for some other viruses such as rhinovirus [34] or mouse hepatitis virus [35].

Taken together, our results may be explained by the observation that HAstV capsid acts as an inhibitor of complement activation [16,17,18], and that intracellular uptake of complement factors bound to viral particles may trigger innate immune responses within cells [15]. Since FBS was added to the post-infection medium in all experiments, it is plausible that the activation of innate responses was delayed due to the action of the HAstV capsid limiting deposition of complement factors on the viral surface. In addition to this, other mechanisms may also contribute to minimize IFN production within HAstV-infected cells. Although active antagonists of the type I IFN response have been characterized for other well-studied gastroenteritis viruses such as rotavirus [32, 33], as reviewed in [19], viral countermeasures to limit cellular antiviral responses may also be achieved by several mechanisms such as tightly controlling virus transcription and replication to minimize the production of dsRNA, encapsidating all forms of viral RNAs produced within the cell, protecting the 5’ end of their RNAs with a cap structure or a VPg protein from recognition by RIG-I, or by replicating within intracellular membrane vesicles to “hide” any viral RNA from the cytoplasmic cellular innate recognition machinery. Recent work in our laboratory has experimentally confirmed that HAstV genome is covalently linked to a VPg protein on its 5’ end [4], and large membrane rearrangements can be observed in HAstV-infected cells, with virus aggregates surrounded by double-membrane vacuoles [5,36]. Indeed, HAstV assembly seems to start at cellular membranes, at the same site where nonstructural proteins replicate the genome. Cell fractionation studies have shown that both positive and negative HAstV RNAs and also nonstructural proteins localize in vesicles where capsid protein gets anchored after synthesis [36].

Despite these strategies to minimize innate antiviral responses, IFN-β would be expressed at the late stages of the viral replication cycle. In addition, exogenous IFN is able to partially reduce viral replication and drug inhibition of IFN signaling enhances virus replication, suggesting that HAstVs may be regarded as IFN-sensitive viruses. Indeed, innate responses have been shown to play a role in controlling AstV replication in available animal models. In turkeys, AstV primary infections are controlled by the expression of inducible nitric oxide synthase (iNOS) and the subsequent increase in its innate immune mediator NO [14], and in mice, AstV replication in the intestine and viral shedding are significantly higher in Stat1-/- animals than in wild-type mice [13]. Both the Jak/Stat1 pathway and iNOS activity have been shown to be important for the control of other viral gastroenteritis agents such as noroviruses [37], which are also sensitive to exogenous IFN [38]. Also recently, type III IFNs (IFN-λ) have gained importance as key mediators of antiviral immunity for intestinal infections [39]. Although IFN-λ activates the same antiviral pathways as type I IFNs, they act through a different receptor that is primarily expressed on the gastrointestinal and other mucosal epithelia. Interestingly, IFN-λ determines the intestinal epithelial antiviral host defense against acute rotavirus infections [40] and persistent infections associated to murine noroviruses [41], and its therapeutic potential has been experimentally proven in mice. Although we still do not know whether IFN-λ may also be critical to control HAstV infections, we have confirmed that synthesis of IFN-λ mRNA takes place within HAstV-infected CaCo-2 cells (data not shown), and future experiments in our laboratory will aim at characterizing this response at the cellular level.

Besides the complete elucidation of the mechanism used by HAstVs to attenuate the cellular type I IFN response, it would also be important to examine whether an interplay occurs between the expression of IFN-β mRNA and the activation of apoptosis. Within CaCo-2 cells, apoptosis has been shown to be required at the late stages of infection for maturation of HAstV capsids and cellular caspases play an active role in the release of virions from infected cells through a non-lytic mechanism [42,43]. Although some motifs found in nsP1a are potential apoptosis inductors [42], apoptosis may also be a consequence of the cellular IFN response. Action of type I IFN within a cell promotes the expression of more than 300 interferon stimulated genes (ISGs), more than 15 of which have pro-apoptotic functions. Additionally, in certain cell types, some of the cellular players in the IFN signaling pathway may also activate caspases and apoptosis, independently of IFN [44,45,46]. In our study, when IFN response was inhibited by BX795 inhibitor, we observed a 2-fold increase in viral RNA and infectious titer, but 4-fold increase in total capsid protein measured by an end-point dilution ELISA assay (Table 1), suggesting that inhibition of IFN allows the virus to replicate to higher titers, but also results in a high proportion of capsids which may not have been properly assembled, encapsidated and/or matured. Although further experiments should be performed, it is plausible that at late stages of infection HAstV may take advantage of the cellular IFN response activation to trigger apoptotic signals and specific caspases required for their late steps of capsid morphogenesis and virion release from the cell.

Finally, we report different levels of IFN-b activation upon infection with HAstV mutants differing in their nsP1a/4 gene. Previous experiments in our laboratory described that different nsP1a/4 variants correlate with differences in viral replication phenotype in cell culture as well as in virus shedding in patients [6,47]. Here we have shown that compared to HAstV-VI and HAstV-XII, HAstV-IV is able to replicate in CaCo-2 cells up to higher titers without inducing such a strong cellular innate response. Although these experiments were done including FBS in the culture media and this could be activating innate responses through complement deposition on viral particles, either input particles or de novo produced particles, our hypothesis is that either directly or indirectly nsP1a/4 genotype influences the level of IFN response induced within cells. Consistent with this idea, viral stocks produced in the laboratory in the absence of FBS for HAstV-IV have higher viral titers than stocks produced for the other genotypes (data not shown), and clinical isolates corresponding to genotype IV have been associated to persistent gastroenteritis [47]. Thus, although the link between nsP1a/4 variability and IFN response may be indirect, our results highlight that variability within nsP1a/4 protein may have clinical implications in terms of pathogenicity and the potential role of nsP1a/4 protein as an antiviral target.
